# Data on wastewater treatment plant by using wetland method, Babol, Iran

**DOI:** 10.1016/j.dib.2017.12.034

**Published:** 2017-12-20

**Authors:** Yousef Dadban Shahamat, Hosseinali Asgharnia, Laleh R. Kalankesh, Mehdi hosanpour

**Affiliations:** a Environmental Health Research Center, Golestan University of Medical Sciences, Gorgan, Iran; b Department of Environmental Health Engineering, Babol University of Medical Sciences, Babol, Iran; c Department of Environmental Health Science, Student Research Committe, Health Sciences Research Center, School of Public Health, Mazandaran University of Medical Sciences, Sari, Iran; d Department of Environment and Energy, West Tehran Branch, Islamic Azad University, Tehran, Iran

**Keywords:** BOD, Babol, COD, Horizontal subsurface flow wetland, TSS, TSD

## Abstract

Date in this paper highlights the applications of constructed horizontal surface flow (HF-CW) wetland with two different local plants (Louis latifoila and Phragmites -*australis (Cav.) Trin*) at the wastewater treatment plant in Babol city. This system was designed as an advanced treatment unit in field scale after the treatment plant. Parameters such as Total Dissolved Solid (TDS), Total Suspended Solid (TSS)**,** Turbidity, Biological Oxygen Demand (BOD) and Chemical Oxygen Demand (COD), were investigated. The result shows that treatment efficiency increases with the passage of time. The efficiency of Phragmites planted setups in open environment was fairly good for all studied parameters (28.6% of TDS, 94.4% for TSS, 79.8% for turbidity, 93.7% for BOD and 82.6% for COD). The efficiency of the *latifoila* set up was also good, but lower than that of Phragmites (26.5% of TDS, 76.9% for TSS, 71.5% for turbidity, 79.1 for BOD and 68.8% for COD). In brief, the obtained dates show that using local plants in (HF-CW) wetland not only effectively reduces various contaminants from the effluent of the wastewater according to *Effluent Guideline regulations* (WHO & EPA), but it is also a cost- effective and environmentally friendly method. Also, it was calculated that in full scale operation [time (1 day) and a depth (0.3 m)], 8 ha of wetland was needed.

**Specifications Table**

**Table:** 

Subject area	*Environmental Engineering*
More specific subject area	*advanced treatment of wastewater*
Type of data	*Table, graph, figure*
How data was acquired	*All data obtained, such as (TDS, TSS**,** Turbidity, BOD, COD), were performed following standard test protocols as presented in Standard Methods for the Examination of Water and Wastewater.*
Data format	*analyzed*
Experimental factors	*These experimental setups were run in an open environment near field scale constructed wetland. Three parallel troughs were built. Two troughs were planted with buds of Louis latifoila and Phragmites, respectively, and the third one was controlled trough. Samples were collected daily and transferred to the laboratory for testing. A total of 120 samples was collected to measure the TSS, TDS, Turbidity, BOD and COD* according *Standard Methods* for the *Examination of Water and Wastewater.*
Experimental features	*The performance efficiency of each setup was studied. For two setups Louis latifoila and Phragmites vegetated efficiency was recorded and compared with control through.*
Data source location	*Babol university of Medical Science, Mazandaran, Iran*
Data accessibility	*Data are accessible with the article*

**Value of the data**

● Data were described with changes in TDS, TSS, Turbidity, BOD and COD in the effluent wastewater of Babol city, by the application of the wetland process.
● Data shows that wetland plants such as *Louis latifoila* and Phragmites, which are native to the north of Iran can be used as a cost-effective source to improve the quality of the treated effluent.
● Data of this study can be used to design the wetland for removal of a wide range of pollutants in wastewater treatment.
● Data are important for the discharge of effluents to the environment, especially resource water, aqueous and agriculture.

## 1 Data

The data in Table 1 show the characteristics as well as the efficiency of *L. latifoila* and Phragmites plants in the removal of contaminants in effluent wastewater during the different Hydraulic Retention Time (HRT). This result can be compared with that of the unvegetated trough.

**Table 1:** Efficiency removal data of the TDS, TSS, Turbidity, BOD and COD by Phragmites, *L. latifoila* and control troughs wetland in the different Hydraulic Retention Time.

**Parameters**		**Control trough**		**Phragmites trough**		**Louis latifoila trough**		**Input**
**HRT (Day)**		**Efficiency%**	**Output**	**Efficiency%**	**Output**	**Efficiency%**	**Output**	
COD^a^	1	61.06±4.89	14.32±2.21	67.56±5.26	11.98±1.95	66.36±5.38	12.35±2.07	36.67±1.55
	3	59.40±7.18	14.50±1.78	69.48±4.06	10.78±1.59	65.75±6.23	12.20±2.04	35.79±3.25
	5	49.94±9.41	14.40±1.95	78.44±3.48	6.36±1.70	68.8±2.55	9.08±1.12	29.20±3.36
								
BOD^a^	1	46.48±13.02	11.62±4.47	75.99±9.64	5.44±3.37	70.24±11.00	6.78±3.93	21.78±7.86
	3	46.62±7.54	11.16±3.94	78.71±5.07	4.64±1.82	74.91±5.07	5.39±1.65	21.70±5.67
	5	56.01±11.68	8.70±3.97	82.59±3.52	3.26±0.72	79.05±3.50	3.95±0.90	18.97±4.16
								
TDS^b^	1	19.35±5.38	466.90±21.78	24.54±5.18	436.60±38.36	21.51±5.46	454.50±24.18	580.10±26.11
	3	18.04±4.57	490.10±39.22	25.81±7.21	436±38.36	22.83±5.99	464.90±30.50	593.3±73.5
	5	25.45±4.38	466.00±19.89		446±34.03	26.47±5.83	459±34.04	625.90±22.25
								
TSS^b^	1	64.00±10.34	7.43±4.44	77.29±9.87	4.52±2.58	75.67±10.7	5.12±2.66	19.60±8.74
	3	60.02±3.59	4.48±1.11	81.58±3.63	1.55±0.82	76.93±8.25	5.51±0.96	11.12±3.55
	5	60.02±3.59	9.37±2.09	79.97±1.91	4.62±0.48	75.21±3.51	5.91±1.81	23.40±4.30
								
Turbidity^c^	1	51.04±5.37	11.17±2.02	78.10±6.22	6.04±2.41	71.45±6.78	6.88±2.61	23.48±6.00
	3	65.40±4.52	7.00±2.19	84.77±4.38	3.14±1.33	82.32±7.27	3.65±1.77	19.92±4.85
	5	75.72±3.64	5.64±1.36	93.66±1.80	1.42±0.29	93.05±1.93	1.56±0.21	23.30±4.35

a (mg L^−1^).

b (g L ^−1^).

c NTU.

**Fig. 1:**
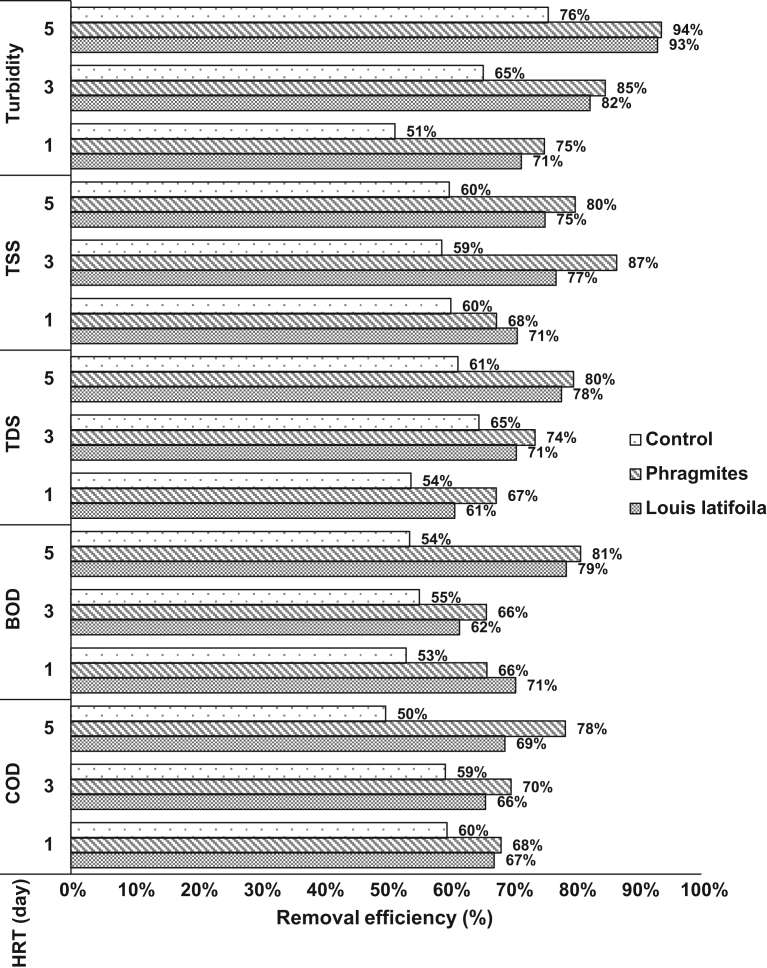
Compare removal efficiency in various vegetation troughs.

Fig. 1 shows the comparison of removal efficiency (TDS, TSS, Turbidity, BOD and COD) at the different troughs (*L. latifolia*, Phragmites and control) during different Hydraulic Retention Time.

## 2 Experimental design, materials and methods

### 2.1 Area of study

Babol is located in the north of Iran, between the northern slopes of the Alborz Mountains and the southern coast of the Caspian Sea (36 °40 N 52 °50E). This research was conducted in the wastewater treatment plant in Babol city. With the rapid increase in the population of this region, it is estimated that 24.5 million liters per day (MLD) of domestic wastewater is generated from urban centers against 0.1 (MLD) of the industrial wastewater. Thus, big gaps are observed in the quality of wastewater treatment in this region.

#### 2.1.1 Design of horizontal subsurface flow, constructed wetland setup

**Fig. 2:**
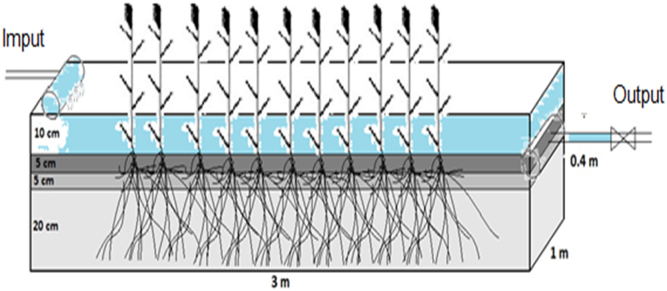
Schematic experimental setups.

**Fig. 3:**
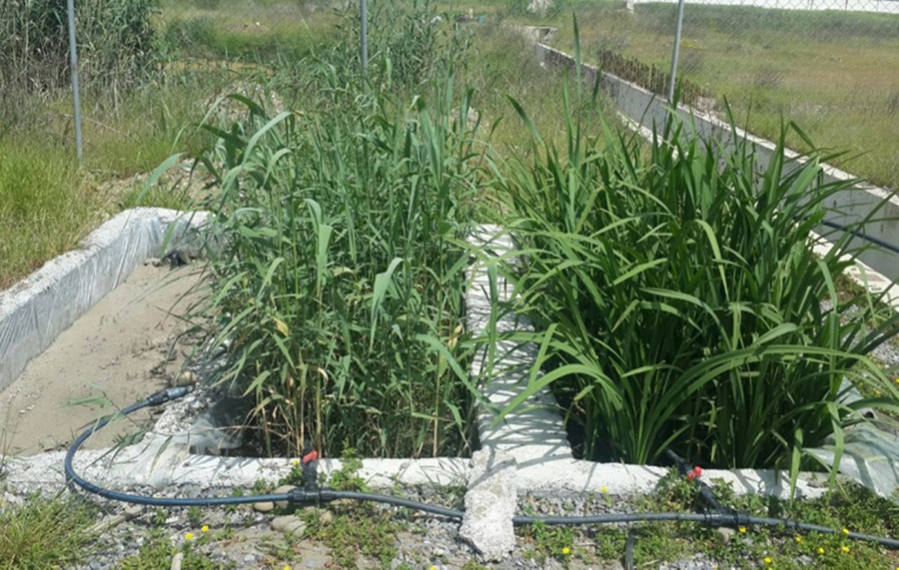
Set up of constructed wetland built in an open field environment.

These experimental setups were run in an open environment using a field scale constructed wetland. Three parallel troughs were built. Two troughs were planted with buds of *L. latifoila* and Phragmites, respectively, and the third one was a controlled trough. In each trough, a concrete pot was used with dimensions of approximately 3×1×0.3 m (length×width×height) and was filled with (pea gravel+tiny sand+coarse sand) 20+5+5 cm, (Fig. 2, Fig. 3). Each trough was arranged sequentially by making interconnections with polyvinyl chloride pipes (1 in.) which were placed in decreasing heights to help the natural flow of wastewater under gravitational pull. In addition, at the entrance each of them was embedded in the pump for adjusting the wastewater remaining time (1, 3 and, 5 days) [1].

#### 2.1.2 Determination of the TDS, TSS, Turbidity, BOD and COD concentration in the effluent wastewater

The performance efficiency of each trough's setup was studied, also two setups *L. latifoila* and Phragmites vegetated efficiency were recorded and compared with the control trough. Setups were operated from the beginning of April to the end of July 2016. The temperature was continuously monitored during the study by using a thermometer and was found to be in the range of 15 to 32 °C. For testing each parameter, samples were collected daily and transferred to the laboratory. A total of 120 samples were collected to measure the concentration of the COD, BOD, TDS, Turbidity and TSS, according to Standard Methods for the Examination of Water and Wastewater [2]. To analyze the data, Statistical Package for Social Science (SPSS) software (version 19) was used.

## Figures and Tables

**Figure:**
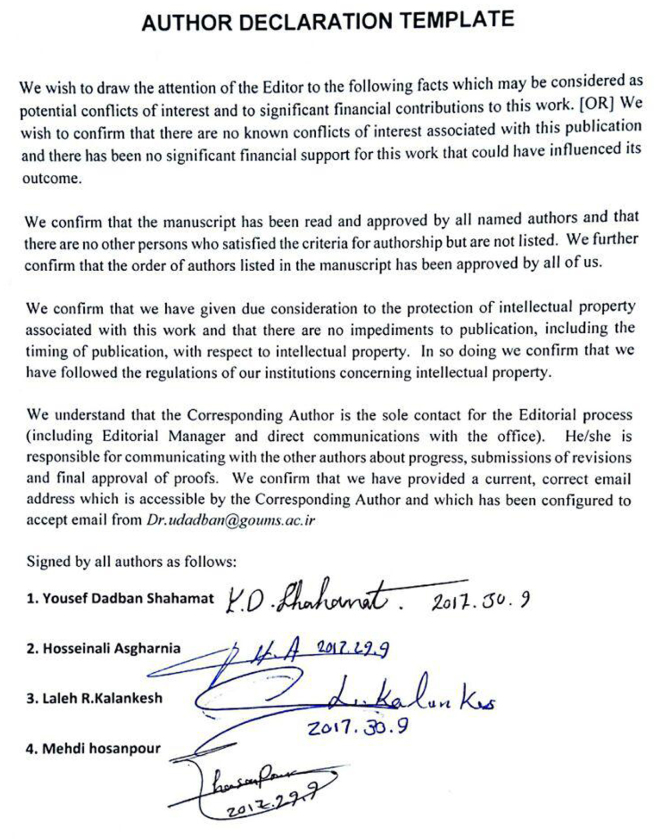

